# World diabetes day 2013: diabetes mellitus and nephrology

**Published:** 2013-07-01

**Authors:** Abdolah Hajivandi, Massood Amiri

**Affiliations:** ^1^Department of Epidemiology and Biostatistics, Bushehr University of Medical Sciences, Bushehr, Iran; ^2^Social Health Determinants Research Center, Shahrekord University of Medical Sciences, Shahrekord, Iran

**Keywords:** World diabetes day, Renal failure, Diabetic nephropathy

**Implication for health policy/practice/research/medical education:**

November 14, world diabetes day is the best opportunity to discuss about different aspects of diabetes mellitus because the incidence of type 2 diabetes mellitus is increasing rapidly. In addition, diabetic nephropathy is a main cause of renal disease. In fact, many more people will be at risk of type 2 diabetes mellitus as well as the fact that about 470 million people globally will be pre-diabetics. Based on the definition of World Health Organization (WHO), people with pre-diabetes have fasting blood sugar (FBS) concentrations between 110 mg/dl and 126 mg/dl or between 101 mg/dl and 124 mg/dl, based on the definition of American Diabetes Association.

World Diabetes Day (every year on November 14) is a good opportunity to emphasis on diabetes and its related aspects and correlation with other health conditions as well as available programs and services for diabetic patients (1-3). Different topics have been discussed in the past years have been diabetes and lifestyle, diabetes, human rights, the costs of diabetes (3-5). In addition, other topics have been focused such as diabetes and foot care in 2005, diabetes in the disadvantaged and the vulnerable in 2006, diabetes in children and adolescents in 2007 and 2008 and diabetes education and prevention is the world diabetes day theme for the period 2009-2013 (1-6). Furthermore, due to its importance, pathological classification of diabetic nephropathy has been published by research committee of the renal pathology society in 2010 which in turn could increase the attentions to control and prevention of diabetic kidney disease. Increased blood pressure levels, hyperglycemia and genetic predisposition may be the main risk factors for the development of diabetic kidney disease. Moreover, diabetic nephropathy is the most common cause of chronic kidney disease which is an international health threat with not fully appreciated mechanism of this health condition (7). Diabetic nephropathy could affect about 40% of type 1 and type 2 diabetic patients, increases the risk of death, especially heart conditions. There is still no uniform classification for diabetic nephropathy (7-9). However, it can be explained by increased urinary albumin excretion (UAE) in the absence of other renal diseases. Indeed, diabetic nephropathy may categorized into stages of: micro-albuminuria (UAE>20 μg/min and ≤199 μg/min) and macro-albuminuria (UAE ≥200 μg/min). Therefore, in type 2 diabetic patients, screening should be done at the diagnosis time as well as the following years after diagnosis (6-8). To achieve the best metabolic control (HbA1c <7%), controlling blood pressure (<130/80 mmHg or <125/75 mmHg if proteinuria >1.0 g/day and increased serum creatinine), using medications with blockade effect on angiotensin II, and controlling hyperlipidemia are the most effective programs to prevent the progression of albuminuria, by making delay in the development of diabetic kidney disease in patients with type 1 and type 2 diabetes. However recently, there was much attention towards better knowledge of morphologic lesions in diabetic nephropathy and proposing a classification for diabetic nephropathy to better control of this disease.

## Conclusion

Classification of diabetic nephropathy may lead to a better communication and collaboration between renal pathologists and clinicians, to prepare a logistic structure for prognostic and interventional researches, and to improve clinical management and improved efficiency. In addition, the application of mentioned pathological classification of diabetic nephropathy may increase the diagnosis rate and raise the attention towards tubular and interstitial injury in diabetic nephropathy, early diagnosis and treatment of diabetic nephropathy (7-12).

## Authors’ contributions

All authors wrote the paper equally

## Conflict of interests

The authors declared no competing interests.

## Ethical considerations

Ethical issues (including plagiarism, data fabrication, double publication) have been completely observed by the authors.

## Funding/Support

None.
